# Mutation spectrum analysis of Duchenne/Becker muscular dystrophy in 68 families in Kuwait: The era of personalized medicine

**DOI:** 10.1371/journal.pone.0197205

**Published:** 2018-05-30

**Authors:** Fawziah Mohammed, Alaa Elshafey, Haya Al-balool, Hayat Alaboud, Mohammed Al Ben Ali, Adel Baqer, Laila Bastaki

**Affiliations:** 1 Medical Laboratory Sciences, Faculty of Allied Health Sciences, Kuwait University, Jabriah, Kuwait; 2 Kuwait Medical Genetic Centre, Ministry of Health, Shouaikh, Kuwait; King Abdulaziz University Hospital, SAUDI ARABIA

## Abstract

Duchenne and Becker muscular dystrophies (DMD/BMD) are X-linked recessive neuromuscular disorders characterized by progressive irreversible muscle weakness and atrophy that affect both skeletal and cardiac muscles. DMD/BMD is caused by mutations in the *Dystrophin* gene on the X chromosome, leading to the absence of the essential muscle protein Dystrophin in DMD. In BMD, Dystrophin is partially functioning with a shorter protein product. Recent advances in molecular therapies for DMD require precise genetic diagnoses because most therapeutic strategies are mutation-specific. Hence, early diagnosis is crucial to allow appropriate planning for patient care and treatment. In this study, data from DMD/BMD patients who attended the Kuwait Medical Genetic Center during the last 20 years was retrieved from a Kuwait neuromuscular registry and analyzed. We combined multiplex PCR and multiplex ligation-dependent probe amplification (MLPA) with Sanger sequencing to detect *Dystrophin* gene mutations. A total of 35 different large rearrangements, 2 deletion-insertions (Indels) and 4 substitution mutations were identified in the 68 unrelated families. The deletion and duplication rates were 66.2% and 4.4%, respectively. The analyzed data from our registry revealed that 11 (16%) of the DMD families will benefit from newly introduced therapies (Ataluren and exon 51 skipping). At the time of submitting this paper, two cases have already enrolled in Ataluren (Tranlsarna™) therapy, and one case has been enrolled in exon 51 skipping therapy.

## Introduction

Duchenne and Becker muscular dystrophies (DMD; MIM 310200/BMD; MIM 300376) are X-linked recessive neuromuscular disorders. DMD is the most common and most severe form, with an incidence of one in 3500–5000 live male births irrespective of race or ethnicity [1].

DMD/BMD is caused by mutations in the *Dystrophin* gene on the X chromosome in the Xp21 region (MIM 300377), which spans 2.4 Mb of genomic DNA. The *Dystrophin* gene is the largest human gene, containing 79 exons that encode a 14-Kb mRNA and produce a 427-Kd membrane protein called Dystrophin [2–3].

Clinically, DMD/BMD is characterized by progressive irreversible muscle weakness and atrophy, primarily affecting skeletal and cardiac muscles. DMD patients are usually diagnosed by the age of 5, wheel chair-dependent before the age of 13 and die from respiratory failure or heart failure by the age of 20. BMD patients, who have a milder manifestation and later-onset of skeletal muscle weakness, are capable of walking independently until the age of 16 years or later and have a normal life expectancy [1]. The clinical differences between DMD and BMD are due to different types of mutations in the *Dystrophin* gene. Most identified mutations are large deletions spanning one or more exons and account for approximately 60–65% of DMD and 85% of BMD mutations [4–7]. The remaining individuals have point mutations, mainly nonsense and frame-shift mutations (10–30%), duplications (5–15%) and intronic or 5`- and 3`-UTR alterations (2%) [2, 4, 5, 7, 8, 9]. Mutations are either inherited from asymptomatic female carriers (∼70%) or de novo (∼30%) [2]. When the mutation causes a shift in the open reading frame (ORF) or generates a premature stop codon, non-functional protein is produced and the patient will suffer from DMD; in contrast, when the reading frame is maintained (in-frame mutation), a partially functional protein is produced and the patients will show the milder clinical symptoms associated with BMD [10–11]. However, the location and extent of in-frame mutations has been found to influence disease severity in Becker patients. Mutations that disrupt the crucial actin-binding domain and/or the binding domain for beta-dystroglycan will result in non-functional Dystrophin and the patients will have the DMD phenotype. Moreover, when there is a large deletion in the central domain (>36 exons), a DMD phenotype generally results [12–13]. Usually, frame-shift mutations will result in a DMD phenotype; however, mutations before exon 8 can present as BMD instead. This has been explained by the presence of alternative start sites in exon 8 that compensate for the earlier mutations. Furthermore, frame-shifting mutations flanking exon 44 generally promote a disease course milder than typical DMD [14–15]. Therefore, it is important to consider the specific mutations associated with DMD when diagnosing DMD patients.

The underlying pathology in DMD patients is the absence of the essential muscle protein Dystrophin. Therefore, new strategies for therapy are aimed at restoring muscle Dystrophin by exon skipping and nonsense mutation suppression [16–17]. Hence, early diagnosis is crucial to allow patients appropriate planning for care and treatment. Herein, we provide a description of *Dystrophin* gene mutations in Kuwait. The study also describes our strategy using a combination of multiplex PCR and multiplex ligation-dependent probe amplification (MLPA) with Sanger sequencing to detect *Dystrophin* gene mutations in DMD/BMD patients who attended the Kuwait Medical Genetic Center (KMGC) during the last 20 years. The study also assessed whether DMD patients are clinically suitable for new genetic therapies according to their mutation type.

## Subjects and methods

### Patients

The KMGC is a tertiary referral center in Kuwait. The KMGC neuromuscular database is the only database available in Kuwait. The database contained 111 DMD/BMD male patients from 68 unrelated families, 20 of which had more than one affected member. These patients attended KMGC from 1996 to 2016. Sixty-eight unrelated male patients, one from each family, were enrolled in this study. From these families, 35 female mothers were also investigated. Kuwaiti families represented 46 (68%) in this series. All patients fulfilled the DMD/BMD diagnostic criteria based on clinical presentation and biochemical testing. Muscle biopsies were completed for 16 (23.5%) of the cases. Sixty-four (94%) were diagnosed as DMD and 4 (6%) were diagnosed as BMD. In most of the patients, diagnosis was confirmed by molecular analysis. The study protocol was approved by the ethical board of KMGC. Written informed consent for genetic study was obtained from each patient or the guardians of individuals <18 years.

### Genomic DNA extraction

Genomic DNA was isolated from 4 ml of EDTA-peripheral whole blood samples using standard phenol/chloroform procedures. The quality and quantity of the DNA was evaluated using a Nanodrop (ND 1000 –BioRad–USA) spectrophotometer and agarose gel electrophoresis.

### Molecular analysis

Multiplex PCR (mPCR) was performed as described by Abbs et al. [18] and Beggs et al. [19] with modification. The modified mPCR protocol tested exons 3, 6, 8, 13, 19, 42, 43, 44, 45, 47, 48, 49, 50, 51, 52, 53 and 60, which cover the hot spot regions in the *Dystrophin* gene. Deletion of a single exon was confirmed by two additional experiments. First, the corresponding multiplex-PCR reaction was repeated. Second, the suspected missing exon and a different exon were amplified in a duplex-PCR reaction for both the patient and healthy male control.

MLPA analysis was implemented on DNA samples from patients who needed further confirmation and for female carrier analysis according to the manufacturer’s protocol (MRC- Holland—Amsterdam—The Netherlands). Sequencing of the all exons and exon-intron boundaries in the *Dystrophin* gene was performed in patients who had no deletions or duplications by the previous two methods.

## Results

### Clinical results

**Fig 1:**
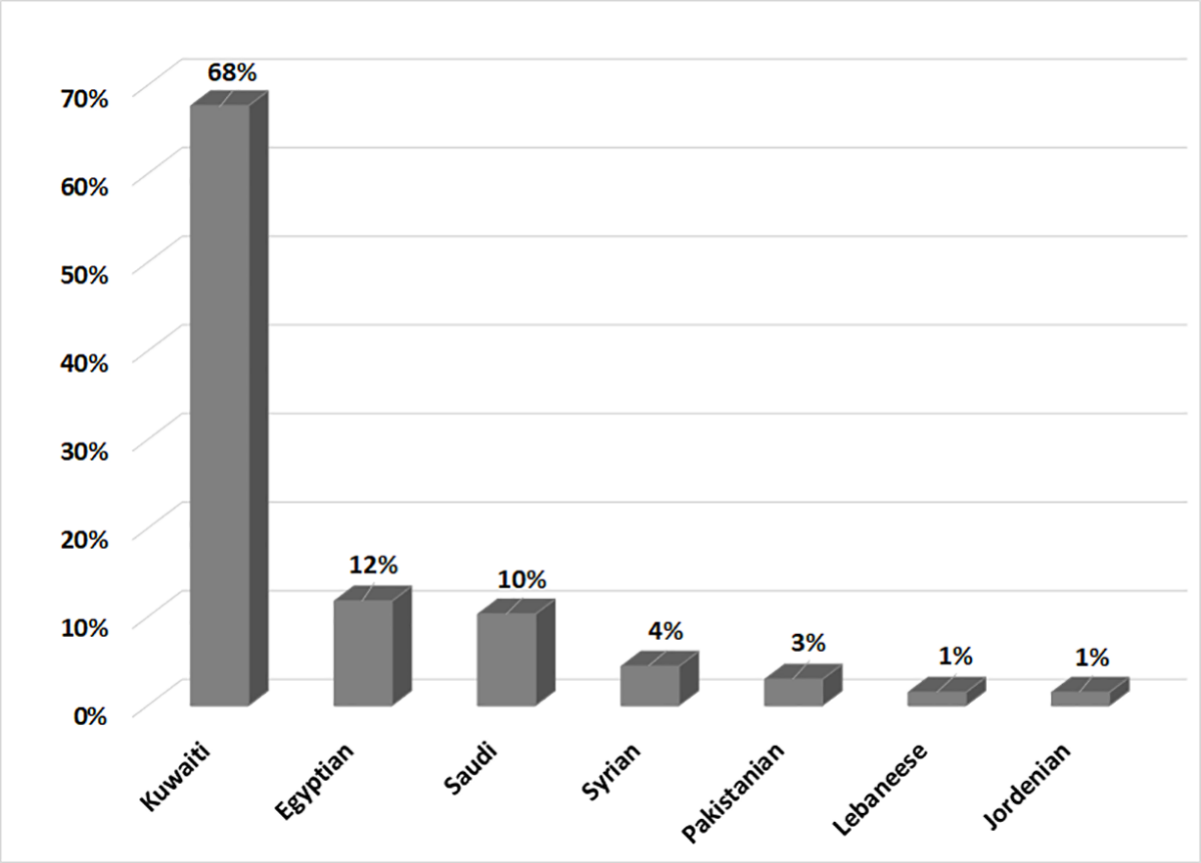
Distribution of DMD/BMD patients according to their nationality.

In this study, we analyzed 68 DNA samples from unrelated patients diagnosed with DMD/BMD based on clinical, biochemical and electrophysiological findings. Kuwaiti patients represented 68% of the sample, followed by 12% Egyptians, 10% Saudi, 6% other Arabs and 3% Pakistani (Fig 1).

The most common presenting symptoms were muscle weakness, frequent falls and abnormal gait. All patients had very high serum CK levels and elevated liver enzymes. The mean age at diagnosis in DMD patients was 2.8±1.2 yrs (1.5–7.0 yrs.). The mean age for wheel chair-dependency was 8.9±1.9 yrs (5.0–13.0 yrs.). The mean age of onset in BMD patients was 7.5±5.3 yrs (2.0–12.0 yrs.). All BMD patients were still ambulant at the time of this study.

Early dilated cardiomyopathy was present in one (1.5%) DMD proband who has two similarly affected older brothers. Muscle biopsies from these patients showed a partial absence of Dystrophin protein. mPCR showed no deletions in this family. No further molecular testing was performed as the family stopped follow-up.

The carrier status of 35 mothers of DMD/BMD patients was examined. Twenty-seven (77.1%) were carriers of *Dystrophin* gene mutations and 8 (32.9%) were non-carriers.

Fourteen (22.2%) of the 64 DMD patients underwent muscle biopsies and showed a complete absence of Dystrophin via immunohistochemical analysis. Two (50%) of 4 BMD patients underwent muscle biopsies and showed mix of weak and absent Dystrophin protein (patchy staining).

### Molecular results

**Fig 2:**
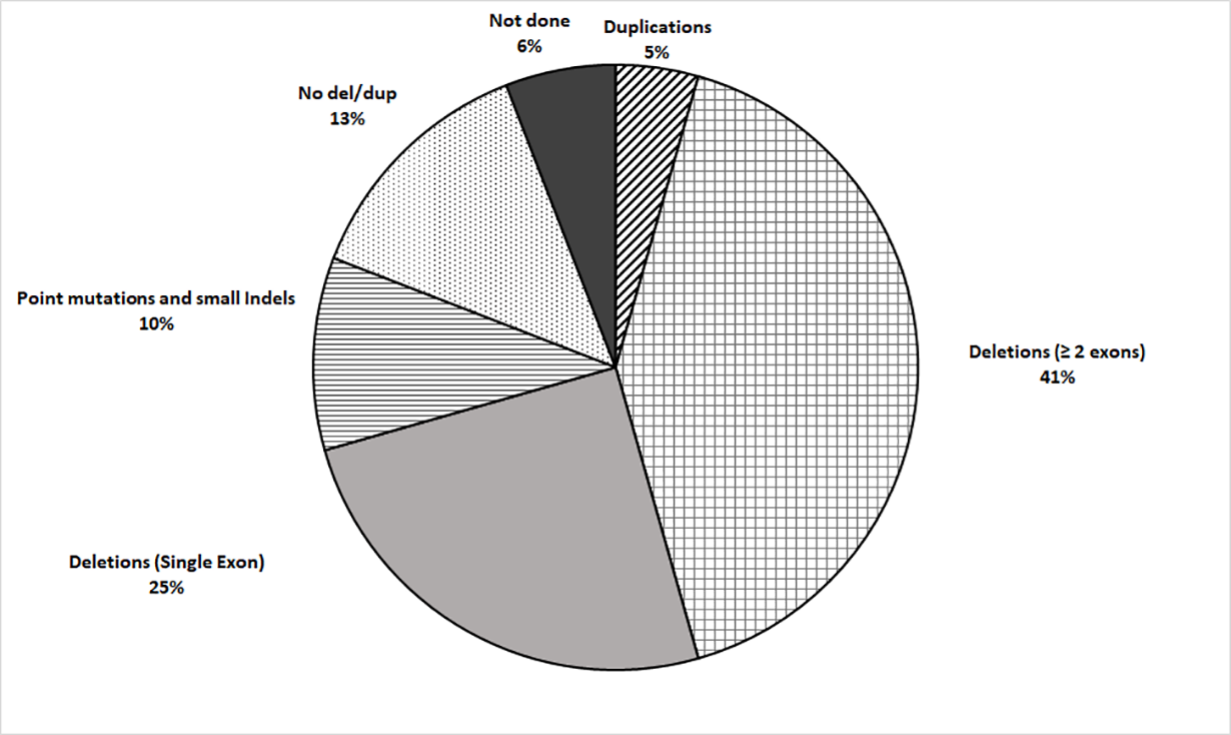
Distribution of mutations in DMD/BMD patients.

**Fig 3:**
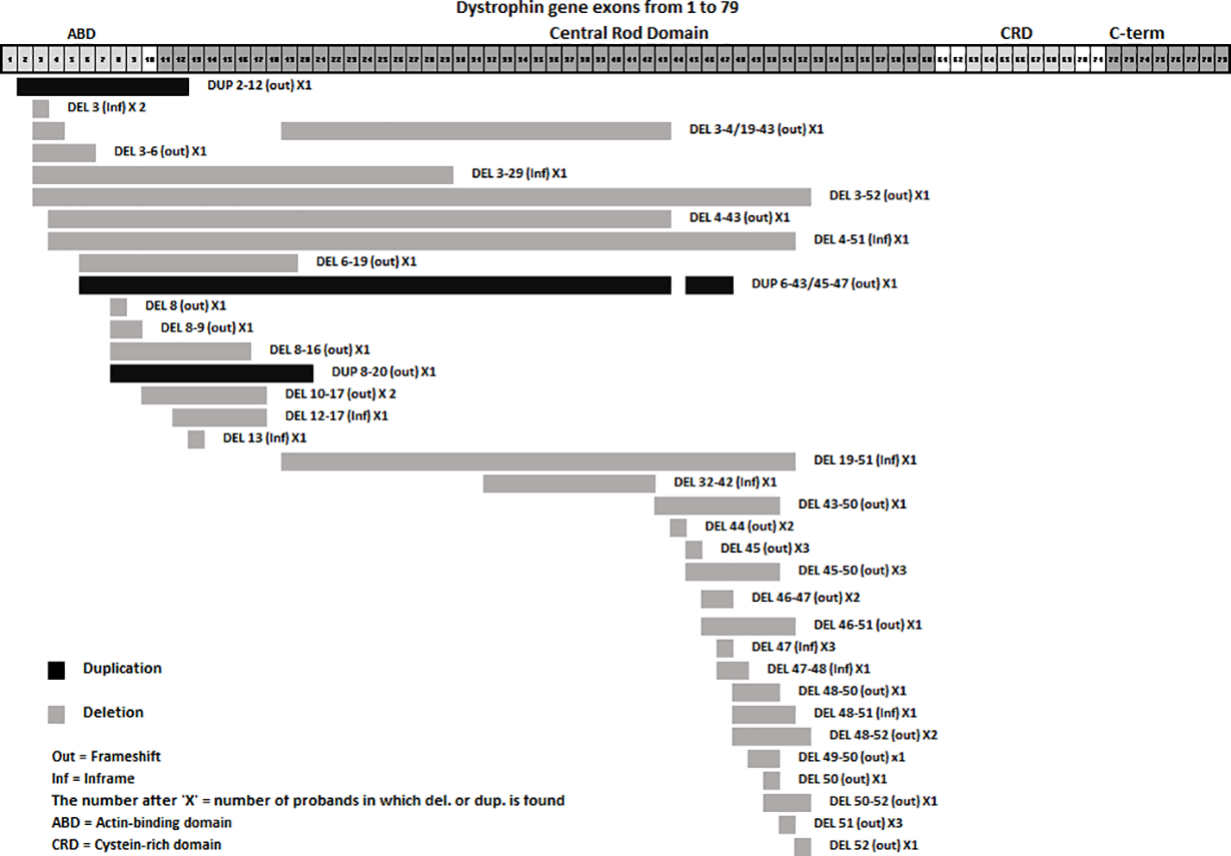
Graphical representation showing the extent of deletions/duplications in the *Dystrophin* gene in DMD and BMD patients. Exon numbers for the *Dystrophin* gene are indicated at the top. The number of cases having similar deletions/duplications is indicated in parenthesis. Deletions and duplications in the *Dystrophin* gene in DMD/BMD patients showing two major hot spots.

Sixty-eight DMD/BMD DNA samples were screened for large rearrangements (one or more exons) in the *Dystrophin* gene by multiplex PCR/MLPA. Forty-five (66%) deletions (42 in DMD and 3 in BMD) and 3 (5%) duplications (all in DMD) were detected (Figs 2 and 3 and Table 1). A single exonic deletion was detected in 17 (37.8%) patients, while two or more exonic deletions were detected in 28 (62.2%) samples. Employing the frame-shift checker from the Leiden Muscular dystrophy website (www.dmd.nl) revealed frame-shift mutations in 34 (75.6%) patients and in-frame mutations in 11 (24.4%) patients.

**Table 1:** Mutation types in DMD/BMD patients.

Clinically approved but no molecular testing, n (%)	No Del/Dup but not tested for point mutation, n (%)	Large deletion (one or more exons), n (%)	Large duplication (one or more exons), n (%)	Substitution mutations & Indels, n (%)	Total, n (%)
**4 (5.9%)**	**9 (13.2%)**	**45 (66.2%); In-frame, 11 (24.4%); Frameshift, 34 (75.6%)**	**3 (4.4%)**	**7 (10.3%)**	**68 (100%)**

Six substitution mutations or Indels were detected in seven unrelated DMD patients (Table 2). Four nonsense mutations created premature stop codons. Two missense variants were detected in one DMD proband. One variant (c.3970C>T (Arg1324Cys)) was most likely pathogenic *in cis* and had a novel (c.7016A>G (His2339Arg)) mutation, which most likely caused a benign variant of unknown significance (VUS). One Indels mutation caused a frame-shift with a premature stop codon. The underlying molecular pathology in 12 DMD and 1 BMD patients has not been determined yet.

**Table 2:** Substitution and deletion-insertion (Indels) mutations in DMD patients.

Substitution mutation & Indels	Mutation type	Number of families	Leiden/HGMD databases
c.1663C>T (p.Q555*)	Nonsense	2	Reported
c.784_786delAAAinsTAG (p.K262*)	Indels; Premature stop codon	1	Novel
c.3702_3710del/c.3701_3702ins CCTT	Indels creating frame-shift	1	Reported
c.5530C>T (R1844*)	Nonsense	1	Reported
c.3970C>T (R1324C); c.7016A>G (H2339R) mostly VUS. These two missense mutations are *in cis*.	Missense	1	Reported; Novel
c.9645C>A (Y3215*)	Nonsense	1	Novel

## Discussion

The KMGC neuromuscular database was recently established to ensure the complete collection of neuromuscular disorders nationwide. In this study, data from DMD/BMD patients was retrieved and analyzed. The mutations in the *Dystrophin* gene among DMD/BMD patients in Kuwait showed high variability. A total of 35 different large rearrangements, 2 deletion-insertions and 4 substitution mutations were identified in 68 unrelated individuals. The deletion and duplication rates were 66.2% and 4.4%, respectively, which is consistent with reports from other parts of the world [8,13,20], except in Taiwanese, Bulgarian and Egyptian populations where the duplication rates were 24.7%, 27% and 27.8%, respectively [6, 21, 22].

Although, deletions and duplications can occur almost anywhere in the *Dystrophin* gene, large deletions and duplications with two hot spots displayed a non-random distribution [13, 19]. In our cohort, of the 45 patients with deletions, 17 (37.8%) had deletions that began in the proximal hot spot (exons 2 to 19). Twenty-seven (60%) had deletions beginning in the distal hot spot (exons 44 to 55). One (2.2%) proband had a deletion that started in exon 32 and extended to exon 42. All three duplications were in the proximal hot spot (5’-prime end of the gene). Our results were in agreement with many previously reported studies, with deletion rearrangements in the DMD gene more likely to be in the distal hot spot and the duplication frequency being highest near the 5’-end of the gene [23, 24].

Mutational analysis of the *Dystrophin* gene in this study revealed non-contiguous complex rearrangements in two unrelated DMD patients. One patient had a deletion and the other had a duplication. The deletion started in exon 3 and extended to exon 43, skipping exons 5 to 18. The duplication started in exon 6 and extended to exon 47, with exon 44 skipped. Baskin et al. [25] reported cases of complex genetic rearrangements with non-contagious deletions and duplications. The increased use of gene-specific high-resolution comparative hybridization arrays (aCGH) in clinical laboratories has enabled the detection of non-contiguous deletions, duplications, and triplications [26]. More work in our laboratories is in progress to characterize the break points in these two cases.

In the present study, we increased the deletion detection rate sensitivity by developing modified multiplex PCR sets according to Abbs et al. [18] and Beggs et al. [19]. This modification allowed for the detection of all the deletions in our cohort. The mPCR results were confirmed by MLPA testing to detect the exact exonic deletion boundaries for all the cases. mPCR showed variations in the *Dystrophin* gene deletion detection rates ranging from 32% to 98% [2]. The deletion rates among Arab, Chinese and North Indian populations were 63.4%, 59.4% and 70.2%, respectively [27–28]. The higher detection rates in some of these studies, including our study, may be related to implementing a multiplex PCR protocol that covers more of the gene than the standard hot spot exons [27, 29]. Based on our study and most of the previously reported studies [19, 30], multiplex PCR is recommended as a first line mutation screening strategy in DMD/BMD patients when there are limited resources because mPCR is a cost-effective, simple and efficient method.

Deletions of exons outside the usual hotspots, duplications and carrier analysis often prevent a precise genetic diagnosis when using mPCR alone. Currently, multiplex ligation-dependent probe amplification (MLPA) has been effectively used for the accurate detection of deletions and duplications in DMD/BMD-affected males and female carriers [20, 31–34]. Using this diagnostic approach, we managed to clarify the disease-causing mutations in 70.6% of DMD/BMD probands and determined the deletion/duplication borders in all the cases. Moreover, MLPA analysis successfully determined the carrier status of 35 mothers. In total, 32% of the mothers were not carriers, indicating de novo mutations in their probands. These data are consistent with the data reported by Nosaeid et al., 2009 [9]. It is worth mentioning that MLPA did not change the positivity of deletion detection among our patients, as we detected all the deleted cases using our modified mPCR. Other studies reported an increase in the deletion detection rate by MLPA in comparison with mPCR [22, 31, 33, 35].

In the present study, 24.4% of DMD patients had an in-frame deletion. Moreover, one BMD patient had a frameshift deletion (del. 48–52). These findings did not fit with the reading frame rule, which suggests that in-frame rearrangements lead to BMD and frameshift mutations lead to DMD. Our results agreed with many previous studies where exceptions to the reading frame rule have been reported [4, 36].

In our cohort, 10% of the DMD patients were due to point mutations and Indels as detected by the direct sequencing method. This percentage is underrepresented and expected to increase because 19% of our patients had not been sequenced yet. Nonsense mutations represented 57% (4 out 7) of the small lesions in our database and 5.8% (4/68) of all mutations. One nonsense mutation, c.9645C>A; Y3215*, detected in this study has not been previously described. Nonsense mutations in the DMD gene have been reported in 10–15% of dystrophinopathy patients [37]. These mutations are expected to promote the premature termination of protein translation and are usually associated with the DMD phenotype. However, some studies have described nonsense mutations that cause the milder BMD phenotype due to altered exon splicing [38, 39].

One (1.5%) missense mutation, c.3970C>T (Arg1324Cys), was detected in a DMD patient in the present study. The low frequency of missense mutations is in accordance with previous publications [37, 39] where missense mutations are rare (0.9%), with most being in the protein-protein interaction domains [36]. Our c.3970C>T (Arg1324Cys) mutation has been described once in the DMD Leiden Open Variation Database (LOVD) (www.lovd.nl). The resulting amino acid substitution Arg1324Cys is within a highly conserved region of the protein and might cause a change in the polarity in this part of the protein that might lead to a change in the Dystrophin secondary and tertiary structures [8]. This substitution is expected to be damaging as predicted by the Pholyphen-2 software analysis. Moreover, this missense mutation is found within exon 29, which is located in the central rod domain of the Dystrophin protein. Several missense mutations have been reported in the rod domain in the literature and in the LOVD [36]. The central rod domain in Dystrophin is expected to act as a linker between the amino-terminal actin-binding domain and the carboxyl-terminal proteins associated with the membrane. The rod domain has been shown to modify the physical properties of lipid membranes [36, 40].

Two (3%) Indels were detected in our DMD patients. One of these, c.784_786delAAAinsTAG (p.Lys262*), was a three-base deletion and a stop codon (TAG) insertion resulting in a premature stop codon and a truncated protein. To the best of our knowledge, this mutation has not been reported before. In contrast, the other mutations (c.3702_3710del/c.3701_3702ins CCTT) have been previously reported in the Leiden Open Variation Database (LOVD; http://www.lovd.nl) and Human Genome Mutation Database (HMGD; http://www.hgmd.cf.ac.uk).

Pathogenic mutations in the *Dystrophin* gene were identified in 81% of DMD/BMD patients in Kuwait. This has allowed for proper diagnosis and counseling and enabled the application of mutation-specific molecular therapies for some of our DMD patients. Recently, multiple novel therapies have been developed, including stop codon read-through agents, exon skipping agents, and utrophin modulators, to target and restore Dystrophin in myocytes from patients suffering from DMD and to offer hope to these patients [41–43]. Our data revealed that 16% of DMD families will benefit from these therapies. Two of our patients with nonsense mutations were already enrolled in the Ataluren (Tranlsarna™) stop codon read-through therapy and a third one is waiting for it, as he is still young. The age and clinical presentations of the remaining patients were not suitable for this therapy. Ataluren (Tranlsarna™) has been developed as a drug to enable the ribosomal read-through of premature stop codons in nonsense mutation DMD (nmDMD) in ambulant patients aged 5 years or older [17, 44]. Its phase 2b study (randomized, double-blind, and placebo-controlled) showed efficacy and safety (well tolerated) after two doses in patients with nonsense mutation dystrophinopathy [45]. However, some safety concerns were identified, such as elevated serum lipids, renal function tests, and blood pressure, when corticosteroids were co-administered [17]. In our patients, the time since the therapy (less than 6 month) was administered is still too short to evaluate these concerns.

Exon skipping with antisense oligonucleotides (AOs) is a promising therapy for DMD that was proposed in 1995 by Takeshima et al. [46] and is currently the focus of clinical trials. The idea is to use antisense oligonucleotides to splice out selected exons from the pre-mRNA, at or next to the mutation site, to generate a translatable transcript from the mutant *Dystrophin* gene [47]. Accordingly, internally deleted but partially functional Dystrophin protein is created from the modified mRNA, similar to what is observed in the milder dystrophinopathy Becker muscular dystrophy [48]. Exondys 51 (eteplirsen), a Sarepta Therapeutics therapy, is the first therapy approved by the FDA for treating DMD patients by exon 51 skipping [49]. Data from our registry revealed that 7 DMD families will benefit from exon 51 skipping therapy. One patient already started the treatment and two are prepared to be immediately enrolled in the treatment program. Four patients may be enrolled later as they are still young.

Clinical trials for AOs targeting exons other than 51 are ongoing and the production of new AOs is being examined in the preclinical stage [50, 51]. It has been estimated that approximately 70% of patients with deletions can be treated by single exon skipping, and this percentage may rise to ∼90% of deletion mutations, 80% of duplication mutations, and 98% of nonsense mutations if multi-exon skipping can be achieved [48, 52, 53].

In conclusion, mutation detection protocols using our modified mPCR as a first line of screening followed by MLPA were simple, rapid, reliable and cost-effective approaches for diagnosing clinically suspected DMD/BMD patients. This approach is recommended in the case of limited resources. The spectrum of mutations detected in the *Dystrophin* gene in our cohort agreed with many internationally reported studies. The presence of an updated DMD/BMD registry in Kuwait helped in assessing patient eligibility for participation in clinical trials and helped detect patients who will benefit from future approved therapies that tackle other exons. Moreover, the Kuwait DMD/BMD registry helped provide better genetic counseling, carrier testing, prenatal screening and PGD testing to families that include a DMD/BMD patient.

## Supporting information

S1 Table List of Primer sequences used in DMD mPCR (Abbs et al., 1991 and Beggs et al., 1990 with some modifications). (DOCX)

S2 Table mPCR mixes and PCR conditions. (DOCX)
