# Supplementary material for: Mutation spectrum analysis of Duchenne/Becker muscular dystrophy in 68 families in Kuwait: The era of personalized medicine
Source: PLoS One. 2018 May 30;13(5):e0197205. doi: 10.1371/journal.pone.0197205 (PMC5976149; doi:10.1371/journal.pone.0197205)
Supplement: S1 Table — (DOCX) [file pone.0197205.s001.Docx]

**S1 Table. List of Primer sequences used in DMD mPCR (Abbs et al., 1991 and Beggs et al., 1990 with some modifications)**

Ex8F: 5’. . gtc ctt tac aca ctt tac ctg ttg ag . .3’

Ex8R: 5’. . ggc ctc att ctc atgttc taatta g . .3’

Ex19F: 5’. . ttc tac cac atc cca ttt tct tcc a . .3’

Ex19R: 5’. . gat ggc aaa agt gtt gag aaa aag tc . .3’

Ex42F: 5’. . cac act gtc cgt gaa gaa acg atg atg . .3’

Ex42R: 5’. . tta gca cag agg tca gga gca ttg ag . .3’

Ex44F: 5’. . gtt gtg tgt aca tgc tag gtg tgt a . .3’

Ex44R: 5’. . tcc atc acc ctt cag aac ctg atc t . .3’

Ex45F: 5’. . ctt tct ttg cca gta caa ctg cat gtg . .3’

Ex45R: 5’. . cat tcc tat tag atc tgt cgc cct ac . . 3’

Ex48F: `5’. . ttg aat aca ttg gtt aaa tcc caa cat g .3’

Ex48R: 5’. . cct gaa taa agt ctt cct tac cac ac . .3’

Ex49F: 5’. . tga tct gca ata cat gtg gag tct c . .3’

Ex49R: 5’. . cac gtc aat ggc aaa tgt aca aca . .3’

Ex51F: 5’. . gaa att ggc tct tta gct tgt gtt tc . .3’

Ex51R: 5’. . gga gag taa agt gat tgg tgg aaa atc . .3’

Ex53F: 5’. . ttg aaa gaa ttc aga atc agt ggg atg . .3’

Ex53R: 5’. . ctt ggt ttc tgt gat ttt ctt ttg gat tg . .3’

PmF: 5’. . GAA GAT Cta gac agt gga tac ata aca aat gca tg. . 3’

PmR: 5’. . ttc tcc gaa ggt aat tgc ctc cca gat ctg agt cc . .3’

Ex3F: 5’. . tca tcc atc atc ttc ggc aga tta a . .3’

Ex3R: 5’. . cag gcg gta gag tat gcc aaa tga aaa tca . .3’

Ex43F: 5’. . gaa cat gtc aaa gtc act gga ctt cat gg . .3’

Ex43R: 5’. . ata tat gtg tta cct acc ctt gtc ggt cc . .3’

Ex50F: 5’. . cac caa atg gat taa gat gtt cat gaa t . .3’

Ex50R: 5’. . tct ctc tca ccc agt cat cac ttc ata g . .3’

Ex13F: 5’. . aat agg agt acc tga gat gta gca gaa at . .3’

Ex13R: 5’. . ctg acc tta agt tgt tct tcc aaa gca g . .3’

Ex6F: 5’. . cca cat gta ggt caa aaa tgt aat gaa . .3’

Ex6R: 5’. . gtc tca gta atc ttc tta cct atg act atg g . .3’

Ex47F: 5’. . cgt tgt tgc att tgt ctg ttt cag tta c . .3’

Ex47R: 5’. . gtc ta acct tta tcc act gga gat ttg . .3’

E60F: 5’. . agg aga aat tgc gcc tct gaa aga gaa cg . .3’

E60R: 5’. . ctg cag aag ctt cca tct ggt gtt cag g . .3’

E52F: 5’. . aat gca gga ttt gga aca gag gcg tcc . .3’

E52R: 5’. . ttc gat ccg taa tga ttg ttc tag cct c . .3’
