# Supplementary material for: Mutation spectrum analysis of Duchenne/Becker muscular dystrophy in 68 families in Kuwait: The era of personalized medicine
Source: PLoS One. 2018 May 30;13(5):e0197205. doi: 10.1371/journal.pone.0197205 (PMC5976149; doi:10.1371/journal.pone.0197205)
Supplement: S2 Table — (DOCX) [file pone.0197205.s002.docx]

**S2 Table. mPCR mixes and PCR conditions**

| **Mix 1 set** | **PCR size (bp)** |  | **Mix 2 set** | **PCR size (bp)** |
| --- | --- | --- | --- | --- |
| Exon 48 | 507 |  | PM3 | 535 |
| Exon 19 | 459 |  | Exon 3 | 410 |
| Exon 44 | 426 |  | Exon 43 | 357 |
| Exon 51 | 388 |  | Exon 50 | 271 |
| Exon 8 | 360 |  | Exon 13 | 238 |
| Exon 45 | 307 |  | Exon 6 | 202 |
| Exon 49 | 282 |  | Exon 47 | 181 |
| Exon 53 | 212 |  | Exon 60 | 139 |
| Exon 42 | 155 |  | Exon 52 | 113 |

**Note:** PCR amplifications were carried out in a 50μl volume comprising approximately 100 ng of template DNA, 10 pmol of each of the 18 primers, a final concentration of 0.4 mM each deoxyribonucleoside triphosphate, and 5 U of Taq Gold DNA polymerase (Amersham Pharmacia Biotech) in 1× PCR buffer supplied by the manufacturer; the MgCl_2_ final concentration in the PCR mixture was adjusted to 2.5 mM. Initial denaturation at 94°C for 7 min was followed by 30 cycles of amplification with 94°C for 30 s, annealing at 65°C for 4 min, and extension at 72°C for 30 s. One cycle of 72°C for 10 min. final extension. Amplification products were analyzed on a 2.5% agarose gel (80 V for 200 min) to separate the different amplification products efficiently.
